# Progress Toward UNAIDS Global HIV Pre-Exposure Prophylaxis Targets: CDC-Supported Oral Pre-Exposure Prophylaxis — 37 Countries, 2017─2023

**DOI:** 10.15585/mmwr.mm7347a3

**Published:** 2024-11-28

**Authors:** Megan E. Peck, Stephanie Davis, Elijah Odoyo-June, Jonathan Mwangi, Elvis Oyugi, Thai Hoang, Marcos Canda, Jessica Seleme, Maria Bock, Lylie Ndeikemona, Sibongile Dladla, Richard Machava, Nyagonde Nyagonde, Abdul Mashauri, Anna Colletar Awor, Stella Alamo, Omega Chituwo, Tina Chisenga, Rickie Malaba, Miriam Mutseta, Carrine Angumua, Kingsly Tse Nkwoh, Janique Ricketts, Kelly-Ann Gordon-Johnson, Victor Adamu, Scott Adamu-Oyegun, John Mondi Benson, Sudhir Bunga, Nasim Farach, Carlos Castaneda, Luis Bonilla, Sharmeen Premjee, Hanna B. Demeke, Gaston Djomand, Carlos Toledo, Ramona Bhatia

**Affiliations:** ^1^Division of Global HIV and Tuberculosis, Global Health Center, CDC; ^2^Division of Global HIV and Tuberculosis, Global Health Center, CDC Kenya; ^3^Ministry of Health, Nairobi, Kenya; ^4^Division of Global HIV and Tuberculosis, Global Health Center, CDC Vietnam; ^5^Division of Global HIV and Tuberculosis, Global Health Center, CDC Mozambique; ^6^Ministry of Health, Maputo, Mozambique; ^7^Division of Global HIV and Tuberculosis, Global Health Center, CDC Namibia; ^8^Ministry of Health and Social Services, Windhoek, Namibia; ^9^Division of Global HIV and Tuberculosis, Global Health Center, CDC South Africa; ^10^Division of Global HIV and Tuberculosis, Global Health Center, CDC Tanzania; ^11^Ministry of Health and Social Welfare, Dodoma, Tanzania; ^12^Division of Global HIV and Tuberculosis, Global Health Center, CDC Uganda; ^13^Division of Global HIV and Tuberculosis, Global Health Center, CDC Zambia; ^14^Ministry of Health, Lusaka, Zambia; ^15^Division of Global HIV and Tuberculosis, Global Health Center, CDC Zimbabwe; ^16^Ministry of Health and Child Care, Harare, Zimbabwe; ^17^Division of Global HIV and Tuberculosis, Global Health Center, CDC Cameroon; ^18^Division of Global HIV and Tuberculosis, Global Health Center, CDC Jamaica; ^19^Division of Global HIV and Tuberculosis, Global Health Center, CDC Nigeria; ^20^Division of Global HIV and Tuberculosis, Global Health Center, CDC South Sudan; ^21^Division of Global HIV and Tuberculosis, Global Health Center, CDC Honduras; ^22^Division of Global HIV and Tuberculosis, Global Health Center, CDC El Salvador; ^23^Division of Global Health Protection, Global Health Center, CDC Dominican Republic; ^24^Division of Global HIV and Tuberculosis, Global Health Center, CDC Dominican Republic.

**Summary**

**What is already known about this topic?**

Pre-exposure prophylaxis (PrEP) reduces the risk for HIV acquisition from sex by 99% and from injection drug use by ≥74% when used as recommended. Globally, 3.5 million persons initiated or continued PrEP during 2023, far fewer than the United Nations Programme on HIV/AIDS goal of at least 21.2 million persons initiating or continuing PrEP globally during 2025.

**What is added by this report?**

During 2017–2023, CDC supported 2,278,743 PrEP initiations in 37 countries, 118.7% of CDC’s overall target for the 7-year period; 856,816 of the initiations occurred in 2023.

**What are the implications for public health practice?**

Additional scale-up is needed to meet the goal of 21.2 million persons using PrEP globally in 2025.

## Abstract

Oral pre-exposure prophylaxis (PrEP) reduces HIV acquisition risk from sex by 99% and from injection drug use by ≥74% when used as recommended. The Joint United Nations Programme on HIV/AIDS (UNAIDS) has set a goal of 21.2 million persons using (initiating or continuing) PrEP globally in 2025. In 2016, CDC, with the U.S. President’s Emergency Plan for AIDS Relief, joined ministries of health to implement PrEP globally. PrEP is beneficial for persons at substantial risk for acquiring HIV, including but not limited to key populations, which include female sex workers, men who have sex with men, persons in prisons and other enclosed settings, persons who inject drugs, and transgender persons. Annual country targets were used to guide scale-up. In 2023, CDC supported 856,816 PrEP initiations, which represents nearly one quarter of the 3.5 million persons globally who either initiated or continued PrEP that year. During 2017–2023, CDC supported PrEP initiations for 2,278,743 persons, 96.0% of whom were in sub-Saharan Africa. More than one half (64.0%) were female and 44.9% were aged 15–24 years. Overall, CDC achieved 118.7% of its PrEP initiation targets for the 7-year period. Among PrEP initiations for key populations, the majority in sub-Saharan Africa were female sex workers, whereas in Southeast Asia, Eurasia, and the Americas, the majority were men who have sex with men. Continued rapid scale-up is needed to meet the UNAIDS goal to end HIV as a public health threat.

## Introduction

Oral pre-exposure prophylaxis (PrEP) reduces HIV acquisition from sex by 99% and from injection drug use by ≥74% when taken as recommended (*1*). In 2015, the World Health Organization recommended that persons at high risk for HIV infection be offered tenofovir-based oral PrEP as part of a comprehensive package of HIV prevention services (*2*). The Joint United Nations Programme on HIV/AIDS (UNAIDS) has set a goal of scaling up services to achieve 21.2 million persons using (initiating or continuing) PrEP globally during 2025 to end HIV as a public health threat by 2030* (*3*). The U.S. President's Emergency Plan for AIDS Relief (PEPFAR), with U.S. government agencies, including the Department of Defense, Agency for International Development, and CDC, provides support for PrEP through partnerships with host countries. Starting in 2016, CDC joined ministries of health in implementing PrEP globally with an initial focus on adolescent girls and young women aged 15–24 years (*4*). In 2020, many PEPFAR-supported countries integrated oral PrEP into their prevention activities. Depending on local HIV incidence patterns, prioritized populations included serodifferent couples (couples in which one partner is living with HIV and the other is not), pregnant and breastfeeding women, adolescent girls and young women, and groups categorized by PEPFAR as key populations. These groups include female sex workers, men who have sex with men (MSM), transgender persons, persons who inject drugs, and persons in prisons and other closed settings (*5*). In collaboration with ministries of health and others, PEPFAR develops annual targets for PrEP initiations for each implementing agency (including CDC) in each country, often based on program performance, financial support, and other factors, including coverage with other HIV interventions such as antiretroviral therapy. CDC supports PrEP programs in four regions including sub-Saharan Africa, Southeast Asia, Eurasia, and the Americas.^†^ This report describes CDC-supported PrEP initiations globally for prioritized populations by region, sex, and age.

## Methods

PrEP initiations from the PEPFAR Monitoring, Evaluation and Reporting Database were analyzed; these were reported semiannually until 2021 and quarterly thereafter, based on the U.S. fiscal year (October 1–September 30) (*6*). The annual number of PrEP initiations was defined as persons enrolled in tenofovir-based oral PrEP during each fiscal year. Annual CDC PrEP initiations during 2017–2023 were described, both overall and disaggregated by region, selected priority population groups (key populations and adolescent girls and young women), sex, and age. These initiations were also compared with CDC PrEP initiation targets to assess percentages achieved. Although programs started reporting PrEP initiations by key population in 2017, initiations by key populations were not reported uniformly until 2019. All analyses were performed using Stata software (version 16; StataCorp). This activity was reviewed by CDC, deemed not research, and was conducted consistent with applicable federal law and CDC policy.^§^

## Results

CDC-supported PrEP initiations in 37 countries increased from 11,397 in 2017 to 856,816 in 2023, approaching approximately 2.3 million total initiations (Table 1).^¶^ Overall, CDC surpassed targets for PrEP initiations (118.7%) for the 7-year period. Annual achievement of CDC PrEP targets ranged among countries from 81.4% to 141.6%. In 2023, all four regions exceeded 100% of their annual target. More than one third (37.6%) of all initiations occurred in 2023.

**TABLE 1:** Annual number of newly initiated CDC-supported pre-exposure prophylaxis users and progress toward CDC targets — U.S. President’s Emergency Plan for AIDS Relief, 37 supported countries, fiscal years 2017─2023*^,^^†^^,^^§^

Region	Fiscal year	PrEP initiations, no. (%)	Annual target	Annual target achieved, %
**Sub-Saharan Africa**	2017	11,302 (0.5)	11,091	101.9
	2018	38,056 (1.7)	32,388	117.5
	2019	68,104 (3.1)	59,583	114.3
	2020	135,775 (6.2)	171,433	79.2
	2021	435,213 (19.9)	457,637	95.1
	2022	678,719 (31.0)	475,293	142.8
	2023	820,777 (37.5)	630,397	130.2
	**All years**	**2,187,946 (96.0)**	**1,989,042**	**119.0**
**Southeast Asia**	2017	95 (0.2)	371	25.6
	2018	227 (0.5)	135	168.1
	2019	3,158 (7.5)	3,083	102.4
	2020	4,457 (10.6)	2,451	181.8
	2021	7,611 (18.0)	13,122	58.0
	2022	12,729 (30.2)	9,577	132.9
	2023	13,900 (33.0)	11,769	118.1
	**All years**	**42,177 (1.9)**	**40,130**	**105.1**
**Eurasia**	2017	0 (—)	—	—
	2018	130 (0.7)	500	26.0
	2019	881 (4.6)	2,097	42.0
	2020	1,273 (6.7)	1,526	119.9
	2021	1,937 (10.2)	2,783	69.6
	2022	6,087 (32.0)	5,439	111.9
	2023	8,741 (45.9)	684	1,277.9
	**All years**	**19,049 (0.8)**	**12,573**	**151.5**
**The Americas**	2017	0 (—)	—	—
	2018	137 (0.5)	150	91.3
	2019	463 (1.6)	799	57.9
	2020	2,836 (9.6)	2,426	116.9
	2021	4,718 (16.0)	4,281	110.2
	2022	8,019 (27.1)	8,414	95.3
	2023	13,398 (45.3)	12,280	109.1
	**All years**	**29,571 (1.3)**	**28,351**	**104.3**
**Global**	2017	11,397 (0.5)	11,465	99.4
	2018	38,550 (1.7)	33,175	116.2
	2019	72,606 (3.2)	65,588	110.7
	2020	144,341 (6.3)	177,323	81.4
	2021	449,479 (19.7)	478,169	94.0
	2022	705,554 (31.0)	498,272	141.6
	2023	856,816 (37.6)	655,058	130.8
	**All years**	**2,278,743**	**1,919,750**	**118.7**

**Abbreviations:** PEPFAR = U.S. President’s Emergency Plan for AIDS Relief; PrEP = pre-exposure prophylaxis.

* U.S. fiscal year = October 1–September 30.

^†^ Not all countries in each region reported PrEP initiations throughout the 7-year period.

^§^ PEPFAR Monitoring, Evaluation, and Reporting data from regions (countries): *sub-Saharan Africa* (Botswana, Cameroon, Côte d’Ivoire, Democratic Republic of the Congo, Eswatini, Ethiopia, Kenya, Lesotho, Malawi, Mozambique, Namibia, Nigeria, Rwanda, South Africa, South Sudan, Tanzania, Uganda, Zambia, and Zimbabwe); *Southeast Asia* (India, Laos, Philippines, Thailand, and Vietnam); *Eurasia* (Kazakhstan, Kyrgyzstan, Tajikistan, and Ukraine); and *the Americas* (Brazil, Colombia, Dominican Republic, El Salvador, Guatemala, Haiti, Honduras, Jamaica, and Panama).

### Annual Number of PrEP Initiations by Region

Nearly all (96.0%; 2,187,946) of CDC-supported PrEP initiations occurred in sub-Saharan Africa, increasing from 11,302 in 2017 to 820,777 in 2023 (Table 1). Throughout the 7-year period, 1.9% of initiations occurred in Southeast Asia, 0.8% in Eurasia, and 1.3% in the Americas. The number of PrEP initiations in all regions combined increased annually, and in each region the largest number of PrEP initiations occurred in 2023. The largest year-to-year increases occurred in sub-Saharan Africa from 2020 to 2021 (220% increase), Southeast Asia and Eurasia from 2021 to 2022 (67% and 214% increases, respectively), and in the Americas from 2022 to 2023 (67% increase).

### PrEP Initiation by Age and Sex

The majority of PrEP initiations in sub-Saharan Africa occurred among females (65.8%), whereas the majority in the other regions occurred among males: Southeast Asia (85.2%), Eurasia (73.8%), and the Americas (77.6%) (Table 2). More than one third (33.7%) of all PrEP initiations were among adolescent girls and young women in sub-Saharan Africa. The largest percentage of initiations occurred among persons aged 15–24 years in sub-Saharan Africa (44.9%) and Southeast Asia (42.9%). The largest percentage of PrEP initiations in the Americas was among persons aged 25–34 years (46.7%) and in Eurasia, among persons aged 35–44 years (31.7%).

**TABLE 2:** Newly initiated CDC-supported pre-exposure prophylaxis users, by sex, age, and key population — U.S. President’s Emergency Plan for AIDS Relief, 37 supported countries, fiscal years 2017─2023*^,^^†^

Region	All PrEP initiations, no. (%)								Key population initiations,^§^ no. (%)					
	Sex		Age group, yrs					Adolescent girls and young women^¶^						
	Female	Male	15–24	25–34	35–44	≥45	Total		Female sex workers	Men who have sex with men	Persons in prisons and other enclosed settings	Persons who inject drugs	Transgender persons	Total
Sub-Saharan Africa	1,436,782 (65.8)**	747,395 (34.2)	993,002 (45.4)	761,841 (34.8)	311,763 (14.2)	121,780 (5.6)	**2,188,386 (96.0)**	767,654 (33.7)	465,696 (63.4)	188,223 (25.6)	26,053 (3.5)	43,903 (6.0)	7,345 (1.0)	**731,220 (90.9)**
Southeast Asia	6,261 (14.8)	35,916 (85.2)	17,990 (42.9)	16,085 (38.4)	5,599 (13.4)	2,243 (5.4)	**41,917 (1.8)**	1,965 (0.1)	1,405 (4.1)	32,187 (93.4)	1 (0.0)	459 (1.3)	635 (1.8)	**34,460 (4.3)**
Eurasia	4,993 (26.2)	14,056 (73.8)	3,633 (19.1)	6,121 (32.2)	6,032 (31.7)	3,250 (17.1)	**19,036 (0.8)**	452 (0.0)	756 (5.5)	9,580 (69.2)	5 (0.0)	3,459 (25.0)	33 (0.2)	**13,833 (1.7)**
The Americas	6,618 (22.4)	22,953 (77.6)	9,301 (31.6)	13,723 (46.7)	4,817 (16.4)	1,563 (5.3)	**29,404 (1.3)**	2,110 (0.1)	4,572 (18.3)	19,315 (77.2)	58 (0.0)	293 (1.2)	766 (3.1)	**25,004 (3.1)**
**Total**	**1,454,654 (64.0)**	**820,320 (36.0)**	**1,023,926 (44.9)**	**79,770 (35.0)**	**328,211 (14.4)**	**12,8836 (5.7)**	**2,278,743 (99.9)** ^††^	**772,181 (33.9)**	**472,376 (58.6)**	**247,387 (30.7)**	**26,117 (3.2)**	**48,049 (6.0)**	**8,675 (1.1)**	**804,517 (35.3)**

**Abbreviations:** PEPFAR = U.S. President’s Emergency Plan for AIDS Relief; PrEP = pre-exposure prophylaxis.

* U.S. fiscal year = October 1–September 30.

^†^ PEPFAR Monitoring, Evaluation, and Reporting data from regions (countries): *sub-Saharan Africa* (Botswana, Cameroon, Côte d’Ivoire, Democratic Republic of the Congo, Eswatini, Ethiopia, Kenya, Lesotho, Malawi, Mozambique, Namibia, Nigeria, Rwanda, South Africa, South Sudan, Tanzania, Uganda, Zambia, and Zimbabwe); *Southeast Asia* (India, Laos, Philippines Thailand, and Vietnam); *Eurasia* (Kyrgyzstan, Kazakhstan, Tajikistan, and Ukraine); and *the Americas* (Brazil, Colombia, Dominican Republic, El Salvador, Guatemala, Haiti, Honduras, Jamaica, and Panama).

^§^ Not all disaggregated indicators were reported throughout the 7-year period. Reporting by key population was not uniform across all countries until 2019.

^¶^ Females aged 15–24 years.

** Data on PrEP initiations by sex in sub-Saharan Africa were missing for some countries in 2017 and 2018.

^††^ Total percentage of PrEP initiations does not sum to 100 because of rounding.

### PrEP Initiation by Key Population

More than one third (35.3%) of all PrEP initiations occurred among persons who were reported to be members of a key population (Table 2). In sub-Saharan Africa, 63.4% of all PrEP initiations among key populations occurred among persons who reported being female sex workers; 25.6% occurred among persons who reported being MSM. In the other three regions, most PrEP initiations among key populations occurred among MSM: Southeast Asia (93.4%), Eurasia (69.2%), and the Americas (77.2%). Across all PrEP initiations among key populations, the lowest proportions occurred among transgender persons (1.1%), persons in prison and other enclosed settings (3.2%), and persons who inject drugs (6.0%). The highest proportion of persons who inject drugs among key populations initiating PrEP occurred in Eurasia (25.0%).

## Discussion

CDC-supported PrEP initiations in 37 countries increased from 11,397 in 2017 to 856,816 in 2023, approaching 2.3 million total initiations. The largest increases were in sub-Saharan Africa, where most PrEP initiations occurred. Despite the impact of COVID-19 mitigation measures, the number of PrEP initiations continued to increase annually in 2020 and 2021, demonstrating programmatic effectiveness in rapidly adapting approaches to virtual and decentralized service delivery models (*7*). The number of PrEP initiations exceeded CDC targets (118.7% overall during 2017–2023). The 856,816 PrEP initiations supported by CDC represent approximately one quarter of the 3.5 million persons globally who either initiated or continued PrEP during 2023 (*3*). However, this number falls short of the UNAIDS target of 21.2 million persons initiating or continuing PrEP in 2025 (*3*). This gap highlights the ongoing need for more investment and innovation in expanding PrEP access worldwide.

Approximately one third of CDC-supported initiations occurred among key populations, an important accomplishment, given the stigma, discrimination, and legal barriers that can make it challenging for these groups to access HIV prevention services (*8*). In sub-Saharan Africa, adolescent girls and young women account for the largest proportion of new HIV infections (*9*) and accounted for approximately one third of CDC-supported PrEP initiations during 2017–2023. In Southeast Asia, Eurasia, and the Americas, most PrEP initiations occurred among MSM, consistent with the epidemiology of new HIV infections in these regions. Globally, the number of initiations among persons in prisons and other enclosed settings, persons who inject drugs, and transgender persons was relatively low, indicating a potential to expand programming among these populations. Alternatives to oral PrEP, including the dapivirine vaginal ring (for HIV risk resulting from sex) and long-acting injectable PrEP, have been introduced; however, implementation has been on a small scale. These options represent an opportunity to expand PrEP programming.

### Limitations

The findings in this report are subject to at least five limitations. First, data quality issues include possible duplication of client records, reporting gaps, or data entry errors; these issues could result in potential over- or underestimation of initiations. Second, because PEPFAR Monitoring, Evaluation and Reporting data do not distinguish between new PrEP users and those who reinitiate at another clinic or under a different name, initiations could potentially be counted more than once. Third, this analysis describes the number of persons who initiated PrEP; however, because oral PrEP effectiveness depends on the degree of adherence, these data alone do not quantify prevention impact. This limitation warrants further analysis. Fourth, the number of PrEP initiations among key populations might be underreported by providers or PrEP users because of stigma, discrimination, and punitive laws. Finally, the number of PrEP initiations presented for each country includes only those supported by CDC, not the total number.

### Public Health Implications

Given the UNAIDS estimate of 3.5 million persons using PrEP globally during 2023, substantial scale-up is needed to reach the 2025 target of 21.2 million persons to reach 2030 goals for ending HIV as a public health threat (*3*). Efforts are ongoing by CDC and others to enhance PrEP use by adapting to users’ evolving needs, such as the demand for more convenient options (e.g., long-acting PrEP). Although PrEP initiations have scaled up substantially in sub-Saharan Africa, potential exists to further expand PrEP access both within the region and beyond PEPFAR-supported countries, particularly considering the increasing HIV incidence in countries outside of sub-Saharan Africa (*3*). Further expansion of PrEP programs that addresses barriers to initiation, including stigma, lack of awareness of PrEP services, and low risk perception among populations at high risk, could increase the number of persons initiating or continuing PrEP. The introduction and implementation of long-acting PrEP products, such as cabotegravir, might accelerate progress toward ending HIV as a public health threat.

## Footnotes

* This goal is also expressed as 10.6 million users throughout 2025, meaning 10.6 million person-years of use. UNAIDS has calculated that if the average duration of PrEP use is 6 months, 21.2 million persons using PrEP during 2025 would be needed to achieve the 10.6 million person-years. This calculation is also sometimes expressed as an approximate UNAIDS 2025 target of 10 million users.

† *Sub-Saharan Africa:* Botswana, Cameroon, Côte d’Ivoire, Democratic Republic of the Congo, Eswatini, Ethiopia, Kenya, Lesotho, Malawi, Mozambique, Namibia, Nigeria, Rwanda, South Africa, South Sudan, Tanzania, Uganda, Zambia, and Zimbabwe; *Southeast Asia:* India, Laos, Philippines, Thailand, and Vietnam; *Eurasia:* Kazakhstan, Kyrgyzstan, Tajikistan, and Ukraine; and *the Americas:* Brazil, Colombia, Dominican Republic, El Salvador, Guatemala, Haiti, Honduras, Jamaica, and Panama.

§ 45 C.F.R. part 46, 21 C.F.R. part 56; 42 U.S.C. Sect. 241(d); 5 U.S.C. Sect. 552a; 44 U.S.C. Sect. 3501 et seq.

¶ Not all 37 countries were supported by CDC during the entire 7-year period, and not all countries reported PrEP initiations annually.
